# Producing co‐production: Reflections on the development of a complex intervention

**DOI:** 10.1111/hex.13046

**Published:** 2020-03-31

**Authors:** Mary Madden, Steph Morris, Margaret Ogden, David Lewis, Duncan Stewart, Jim McCambridge

**Affiliations:** ^1^ University of York York UK

**Keywords:** alcohol, community pharmacy, complex interventions, co‐production, medicines review, patient and public involvement

## Abstract

**Background:**

Patient and public involvement and co‐production are widely used, but nevertheless contested concepts in applied health research. There is much confusion about what they are, how they might be undertaken and how they relate to each other. There are distinct challenges and particular gaps in public involvement in alcohol research, especially when the study focus is on health matters other than alcohol dependence.

**Objective:**

To explore how patient and public involvement and co‐production have been interpreted and applied within a multi‐disciplinary research programme in the development of a complex intervention on alcohol and medicine use in community pharmacies.

**Design:**

The paper presents the authors' critical reflection on a grounded example of how public involvement concepts have been translated into practice in the intervention development phase of a publicly funded research programme, noting its impact on the programme to date.

**Discussion:**

Co‐production adds another layer of complexity in the development of a complex intervention. The research planning requirements for publicly funded research circumscribe the possibilities for co‐production, including impacting on the possibility of stability and continuity over time.

## 1 INTRODUCTION

This paper is co‐authored by researchers and two patient and public involvement (PPI) group members from the National Institute for Health Research (NIHR)‐funded Community pharmacy: Highlighting Alcohol use in Medication aPpointments‐1 (CHAMP‐1) 5‐year research programme. The aim of NIHR Programme Grants for Applied Research (PGfAR) is to deliver research findings that will lead to clear and identifiable patient benefits. As part of this, the NIHR expects active involvement of patients and the public in the research activities of the programme, working with the research team, and not just their inclusion as ‘subjects’ of the research. Through critical reflection on a grounded example, the intervention development phase of this programme of applied health research, the paper identifies some of the challenges and benefits of operationalizing co‐production and PPI in developing a complex intervention which features the sensitive topic of alcohol consumption. The paper examines how PPI and co‐production concepts have been interpreted and applied from the pre‐application stage to the embedded research studies conducted for intervention development. The paper bridges a knowledge gap between funder calls for PPI and co‐production and clarity on how to apply such principles in practice. While acknowledging the considerable challenges, the authors make a case for the importance of including patients and the public in the production of alcohol, and complex interventions research.

## 2 BACKGROUND

### 2.1 The shift from PPI to co‐production

The NIHR in England and the Netherlands Organisation for Health Research and Development (ZonMW) were identified as having, ‘the most extensive involvement of members of the public’ in a survey conducted by authors concerned about avoidable waste in the production and reporting of research evidence.^1^ The authors argue that public involvement in research matters because public funding of research does not relate well to disease burden, certain areas get disproportionate amounts of attention, there is poor transparency, and most studies conducted ignore questions and outcomes of importance to patients, carers and health professionals leading to ‘avoidable waste.’^2^ Others have argued that decisions about research priorities need to involve a greater diversity of perspectives because the pharmaceutical and biotechnology sectors have dominated the health research agenda, and research on the social, environmental, digital and behavioural determinants of health required to improve National Health Service (NHS) services, public health and social care has been under‐resourced.^3^

The NIHR PPI involvement imperative has, unsurprisingly, led to an increase in the levels of PPI activity in NIHR‐funded studies, followed by calls for better reporting in order to achieve an empirically and theoretically informed understanding of the types, quality and impacts of the involvement activity being produced.^4^, ^5^, ^6^ The extent, quality and impact of the reporting of PPI have been inconsistent, and understandings and practices of PPI have been criticized as tokenistic and ‘top‐down.’^5^, ^7^

A 2015 strategic review of NIHR PPI suggested co‐production, ‘as a means of evolving and improving public involvement in research,’ recommending the adoption of co‐production principles to foster partnership, reciprocity and openness.^8^, ^9^, ^10^ The review identified the potential for co‐production to add value, but there has been uncertainty about the meaning of the concept, and confusion about how to actually organize and deliver it in practice.^11^ INVOLVE, the advisory group funded by the NIHR to support active public involvement, published guidance as, ‘a first step in moving toward clarity about what we mean by co‐producing research,’ (p6). This quickly sparked requests to clarify how to turn co‐production theory into action.^12^ The guidance defines co‐production as an approach in which:

… researchers, practitioners and the public work together, sharing power and responsibility from the start to the end of the project, including the generation of knowledge. The assumption is that those affected by research are best placed to design and deliver it and have skills and knowledge of equal importance.[10]

This assumption is clearly important and contains a number of elements, and thus deserves to be examined carefully. Paylor and McKevitt point out that the NIHR framing of co‐production presents it as a more collaborative and egalitarian mode of involvement. They caution, however, that any such potential has to be understood in relation to the prevailing methodological and managerial constraints of funded health research.^13^ The traditions of participatory action research and co‐operative inquiry, in which co‐production methods have evolved over time, are at some considerable remove from those of contemporary applied health research where the randomized controlled trial (RCT) is methodologically dominant for intervention evaluation purposes, and where qualitative research per se has been considered low priority.^14^ There has been little research on how researchers and others involved have responded to the imperative for more participatory knowledge production and how this translates into practice in complex research programmes within a contemporary academic context, ‘influenced by power and incentives.’^15^

### 2.2 What exactly is co‐production in health research?

The term co‐production has its origins in work on urban governance in the United States in the late 1970s which was influenced by Lipsky's work on ‘street level bureaucrats.’^16^ In an early paper aimed at sharpening the definition of the concept, Parks et al^17^ describe co‐production as the, ‘mixing of the productive efforts of regular and consumer producers.’ They drew on the work of Garn et al^18^ to argue that public services are to some extent inevitably co‐produced because of the ways in which people work with, take up and adapt them. For example, policing relies on consent, co‐operation and information from the communities it serves:

… the person being served (the client or consumer) is inevitably part of the production process, if there is to be any production whatsoever. Therefore, the resources, motivations, and skills brought to bear by the client or consumer are much more intimately connected with the level of achieved output than in the case of goods production. The output is always a jointly produced output.[19]

Ostrom identifies co‐production as a process of production, contributed to by people who are not in the same organization, which involves the transformation of a set of inputs into outputs. She describes the co‐production process as one in which, ‘synergy between what a government does and what citizens do can occur.’^19^ She also acknowledges the challenge of engineering such a process:

Designing institutional arrangements that help induce successful coproductive strategies is far more daunting than demonstrating their theoretical existence.[20]

Such challenges, and the origins of the concept of co‐production in contexts far from health research, may carry implications for applications in this area. Despite the positive framing by the NIHR, and the aspirational intent of the INVOLVE definition, Oliver et al^20^ recommend a cautious approach to co‐production in contemporary health research because it carries a ‘dark side’ manifested in practical, personal and professional costs to participants.

The NIHR recommends the use of ‘4R’ criteria as the starting point for reporting on the impact of PPI on research: reach; relevance; refinement and improvement and relationships.^8^, ^21^ Reporting here is guided by the GRIPP2 checklist^4^ and will include the 4R aspects, with a particular focus on ‘refinement and improvement,’ that is the added value that public involvement brought to the first phase of the CHAMP‐1 programme which used a ‘bottom‐up’ approach to co‐producing a complex intervention.

## 3 DEVELOPING A COMPLEX INTERVENTION IN A CHALLENGING TOPIC AREA

**Figure 1:**
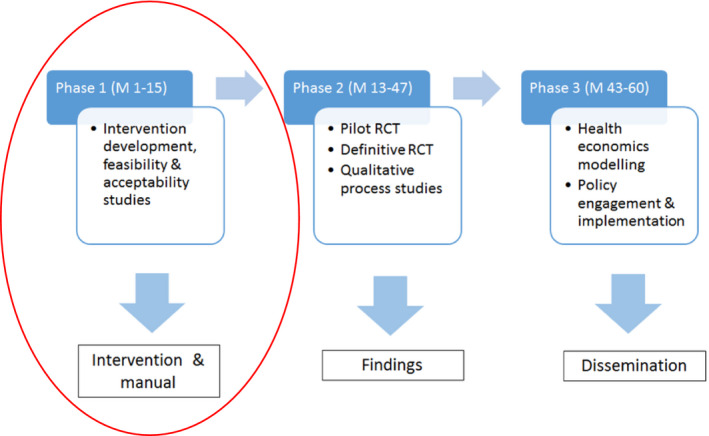
Community pharmacy: Highlighting Alcohol use in Medication aPpointments (CHAMP)‐1 research programme overview

The CHAMP‐1 programme is co‐producing with the pharmacy profession and with patients and carers, a complex intervention for discussion of alcohol within community pharmacy‐based medication reviews to be evaluated in a definitive RCT. This paper focuses on the objective for the first phase of the 5‐year programme and development of the intervention for use within established pharmaceutical services (Figure 1). Below, we explain why the sensitive nature of alcohol discussion makes such an intervention, and the process of its production, ‘complex’ and why the decision was made to ‘co‐produce’.

### 3.1 Alcohol as a sensitive subject

The majority of people have some experience of alcohol because it is very widely used and because its use impacts on people who do not drink alcohol themselves.^22^ For many, alcohol is associated with positive aspects of life, but alcohol is also linked to poor health in various and complex ways, including interactions with medicines, 23 and it is implicated in social problems. It presents a degree of risk for all who use it.^24^, ^25^ Yet, alcohol as a broad population health issue, outside of addiction services, is not an area with a substantive record of public involvement.^26^

Alcohol research has focused predominantly on particular populations and forms of problematized drinking that have stereotypical and highly stigmatized and stigmatizing foundations.^27^, ^28^, ^29^ It is also an area in which alcohol industry agendas are promoted heavily through marketing and social aspects organizations.^30^, ^31^ In keeping with the avoidable waste agenda, the CHAMP‐1 programme is building on an important body of pre‐existing research,^2^ but, given the limited public discourse on alcohol health harms, is not an issue likely to be readily identified by the community/the public.

In a previous alcohol intervention trial in community pharmacy, people who considered their drinking non‐problematic did not consider the proposed intervention or the research topic to be relevant to them, largely because of the ways they thought about the nature of alcohol problems.^32^ Key challenges then for the CHAMP‐1 programme are the lack of perception of personal health hazards due to alcohol outside of stereotypical conceptions of alcohol dependence and the sensitivity of any discussion about alcohol because of the stigmatization of alcohol‐related problems.^28^, ^33^ This sensitivity in talking about alcohol is linked to a sense of vulnerability to negative judgement. Alcohol research is a political arena in which science and public involvement and engagement can be skewed by vested commercial interests,^34^ and where improving communication of risk is key. Therefore, public advisors without vested commercial interests and with an openness to consider varying forms of alcohol harms can potentially make a particularly valuable contribution*.*

### 3.2 The complex intervention context

The approach to intervention development taken in the CHAMP‐1 programme was strongly informed by the investigators' experience of a previous community pharmacy trial of brief alcohol interventions delivered by community pharmacists and the lessons learned in that study.^35^ In short, brief interventions that may be effective elsewhere, and there are grounds for uncertainty about their effectiveness (ie performance in ‘real‐world’ conditions) rather than efficacy (performance in research contexts),^36^, ^37^ were found not to work in the community pharmacy setting, probably because they placed demands on pharmacists to operate beyond the context of pharmaceutical practice.^32^, ^35^ It was considered therefore to be more appropriate to integrate discussion of alcohol use into routine pharmaceutical practice, hence the rationale for a focus on medication reviews. The decision to co‐produce the intervention with patients and with pharmacists was a fundamental change in orientation to intervention development from the previous trial.

There was no one theory‐based ‘off‐the‐shelf’ intervention either available or likely to be effective, or judged adaptable to this context. Patients who participate in medication reviews live with long‐term conditions. Building on previous experience and the input of patient advisors and a lay co‐applicant, the application for NIHR programme funds thus proposed that the experiences and ideas patients have about these conditions and their treatment and management are crucial to understanding their receptivity to alcohol discussions. Drinking alcohol was viewed as posing a potential problem directly via its impact on health and well‐being, and indirectly by potentially reducing adherence to, or the effectiveness of, pharmaceutical treatments.

The aim of the intervention is to help patients consider their own circumstances and make decisions about their own alcohol consumption and medication use. In order to achieve this, it aims to help pharmacists to develop skills and their practice in order to become more person‐centred in consultations. This provides for an intervention opportunity with patients which might in turn help them to impact on their alcohol and medication management. The causal chain involves a series of steps, which may be accomplished more or less well in different interactions, and is therefore complex. It is difficult to be precise about the ‘active’ ingredients of an interaction between a community pharmacist and a person in a medicines review, and how this might relate to other factors to produce behaviour change and subsequent health outcomes for patients.^38^ The intervention developed therefore has to be flexible enough to adapt to the complexities of the systems in which it will be introduced.^39^

People have volition, their thinking, feelings and behaviour can be unpredictable and the contexts in which they live and interact are dynamic, which means many factors can affect outcomes. Community pharmacists and community pharmacies will vary in how receptive they are to such attempts at introducing innovations. There are guidelines on research involving complex interventions from the Medical Research Council which are currently being updated because of on‐going developments in the field.^40^ Informed by these guidelines, our funding application outlined a range of intervention development, feasibility and acceptability studies to prepare the intervention and trial design. The funded programme processes of intervention development and testing have been developed to fit pharmacy routine practice and require patient and public advisors to work with pharmacists and a multi‐disciplinary research team using multiple methods within tight deadlines in a health system engaged in on‐going change.^41^ A programme PPI group, a pharmacy professional practice group (PPPG) and a policy advisory group (PAG) all advise on the design and conduct of the research and are represented on the programme management group.

### 3.3 Intervention development overview

**Figure 2:**
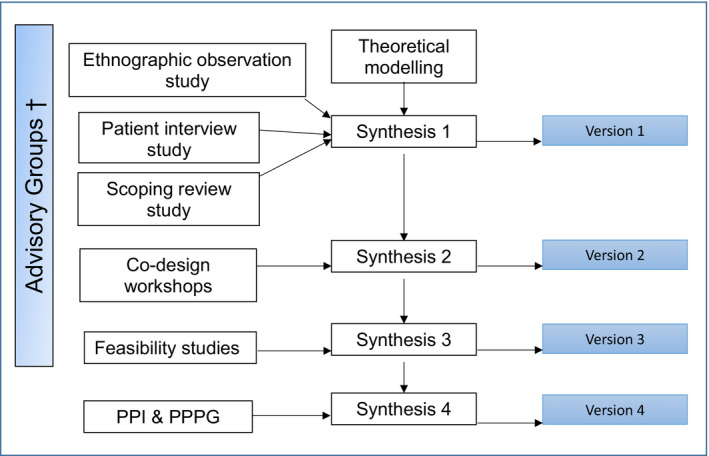
Community pharmacy: Highlighting Alcohol use in Medication aPpointments (CHAMP)‐1 phase 1 intervention development process (versions 1‐4). ^†^Policy Advisory Group (PAG), Patient and Public Involvement Group (PPI), Pharmacy Professional Practice Group (PPPG)

The intervention development phase lasted 15 months and comprised a series of stages to develop versions of the intervention iteratively (see Figure 2). Phase 1 studies received NHS Health Research Authority research ethics approval (Yorkshire & the Humber—South Yorkshire Research Ethics Committee REC reference 17/YH/0406).

Each stage was concluded with ‘synthesis’ discussions by an intervention development subgroup of the wider research team informed by PPI, PAG and PPPG meetings. Qualitative interview and ethnographic observation studies,^33^, ^42^, ^43^, ^44^ and a scoping review of the literature on the particular services being studied^45^ informed the development of the first prototype of the intervention (version 1). Interactive co‐design workshops with patients and pharmacists were arranged to inform a review of this version (producing version 2). Then, a study of how version 2 was conducted by pharmacists in practice informed version 3. Further consultation with the PPI and PPPG groups led to the production of version 4. Drawing on the CHAMP‐1 PPI impact log, the process of establishing a PPI group and its particular input into refining and improving the research studies in the first phase of the programme is reported below.

## 4 PPI IN THE CO‐PRODUCTION PROCESS

### 4.1 Establishing a PPI group

Patient involvement was initiated before drafting of the CHAMP‐1 funding application commenced in a preliminary process. PPI began with a discussion about pharmacists and alcohol advice on an existing online alcohol forum, followed by consultation with members of an established pharmacy PPI group. Co‐author Margaret Ogden (MO) was then recruited as a lay co‐applicant via the NIHR/INVOLVE People in Research website (peopleinresearch.org). Three research team members, including MO, held a PPI event with five people with chronic conditions to further inform the development of the application prior to submission of the grant application (one person continued their membership after funding was awarded). The existing pharmacy PPI group was then consulted again about potential PPI roles and recruitment processes, with an invitation to join the CHAMP‐1 group. The full application was originally submitted approximately 17 months before the research programme actually began.

Once the programme was funded, the lay co‐applicant became Chair of the PPI group, and PPI group recruitment was re‐initiated via existing networks and support groups for long‐term conditions (at whom medicine review services are targeted). We specified that applicants have experience of managing medication for a long‐term condition(s) and regular engagement with pharmacies. Other requirements were experience of drinking alcohol, and the willingness and ability to discuss alcohol and work as part of a group. Some members also bring valuable carer perspectives. Quoting directly from MO who was a co‐applicant and is now a co‐investigator and Chair of the PPI group:

Recruitment of the PPI members has been carefully considered. The group developed gradually. Criteria for membership was defined specifically to reflect and closely mirror the research participant group. I believe this was crucial and has been pivotal to the project.

Recruitment was continuous (and remains so), reflecting changes in members' health and transport problems. By the end of the first phase of the programme (15‐month duration), there were 10 active members of the PPI group (including the Chair) who regularly attended meetings. The demographics of the current group at the end of phase one is shown in Table 1.

**Table 1:** Patient and public involvement (PPI) group demographic data (including PPI chair)

Demographic characteristic	No. of members (total n = 10)
Sex	
Men	6
Women	4
Age range (y)	
45‐54	3
55‐64	3
65‐71	2
Prefer not to disclose	2
Higher education	
Secondary education	3
Higher education post‐16	4
Undergraduate degree	2
Prefer not to disclose	1
Employment status	
Retired	6
Semi‐retired	1
Sickness, disability or unemployment benefits	2
Employed part‐time	1
Ethnicity	
White British or Irish	9
British South Asian	1
Sexuality	
Heterosexual	8
Homosexual	1
Prefer not to disclose	1
Partner status	
Married or partner	7
Divorced	1
Single	2
Alcohol frequency	
4 or more times per week	3
2‐3 times per week	6
2‐4 times per month	1
No. of medications	
8+	1
6‐7	4
4‐5	2
2‐3	2
1	1
Experience of medicines review (MUR/NMS)	6

Patient and public involvement role descriptions and skills training provisions were developed when research staff were in post to support the process. PPI group members requested training to help develop their understanding of the research methods used in the programme. This is contract research, and there have been some changes in staff in the first phase of the programme and some gaps between appointments. PPI continuity has been provided by the Chair of the PPI group (MO) and the Programme Co‐ordinator and co‐author Duncan Stewart (DS). MO and co‐author and representative from the PPI group, David Lewis (DL), have decision‐making powers as members of the programme management group, which also includes pharmacists within the research team, along with non‐pharmacist researchers. This group is chaired by the Principal Investigator and co‐author Jim McCambridge (JM), and it oversees and is responsible for the conduct of the research. DL has found attending and contributing to these meetings more of a challenging experience than the more informal and interactive PPI meetings. In DL's own words:

Opportunities to establish working relationships with people on the programme management group are limited because many members travel from other parts of the country or attend remotely by conference call. There is limited opportunity for pre‐meeting preparation because of the hectic pace of the programme.

In addition to these programme structures, the NIHR requires that the programme is overseen by an independent Programme Steering Committee (PSC), which meets at least annually to supervise and provide expert advice independent from the investigators on behalf of the NIHR and the NHS research sponsor. A member of the PSC is recruited (independent of the PPI group) to represent the interests of patients and the public.^46^

The INVOLVE co‐production ideal of researchers and lay members working together from the start to the end of the project like this is contingent on the stability of people's health, staff continuity and time to support the development of the PPI group while meeting other research targets. The potential to develop relationships and share power and responsibility is constrained within programme management arrangements that need to meet the requirements of the funder and the university and comply with research governance procedures. Decisions about the design of the research need to be made long before funding is awarded.

### 4.2 PPI group role and working methods

The design of CHAMP‐1 working PPI and co‐production processes were informed by previous researcher and lay investigator experiences of PPI and participatory research methods.^47^ The role of the PPI group is to provide a patient perspective throughout the life of the programme by advising the research team on the content and conduct of the research. Outcomes of this are recorded in a PPI impact log (see Appendix 1). The group met four times in the intervention development phase of the programme and is scheduled to meet twice a year thereafter. The content of the meetings is responsive to the research, and meetings are timed accordingly. Each member is paid for attending and preparing for each meeting, and travel expenses are reimbursed. Members are paid at the same sessional rate for their work assisting in research preparation and delivery outside meetings. The PPI Chair and lay co‐investigator views opportunities for deeper involvement in research development as the most innovative part of the programme from her perspective. Quoting MO directly:

The testing out of topic guide and interviews by the PPI members [see below] has been the most innovative and inclusive mechanism of the PPI to date. It went beyond traditional channels of PPI such as reviewing and contributing at meetings. It presented PPI members with opportunities to develop new skills – important for their personal development.

On‐going waves of PPI recruitment present a challenge as experience, and familiarity with a complex programme is lost but also mean that the programme has benefited from ‘fresh’ perspectives over time. The programme has sought to engage members in meaningful rolling dialogue. This has been facilitated at meetings and also by researcher‐led email contact and updates between meetings to maintain momentum and an email discussion forum where members post relevant news about the research topic.

### 4.3 Focusing on issues and dilemmas that arise for patients in medication and alcohol discussions

**Figure 3:**
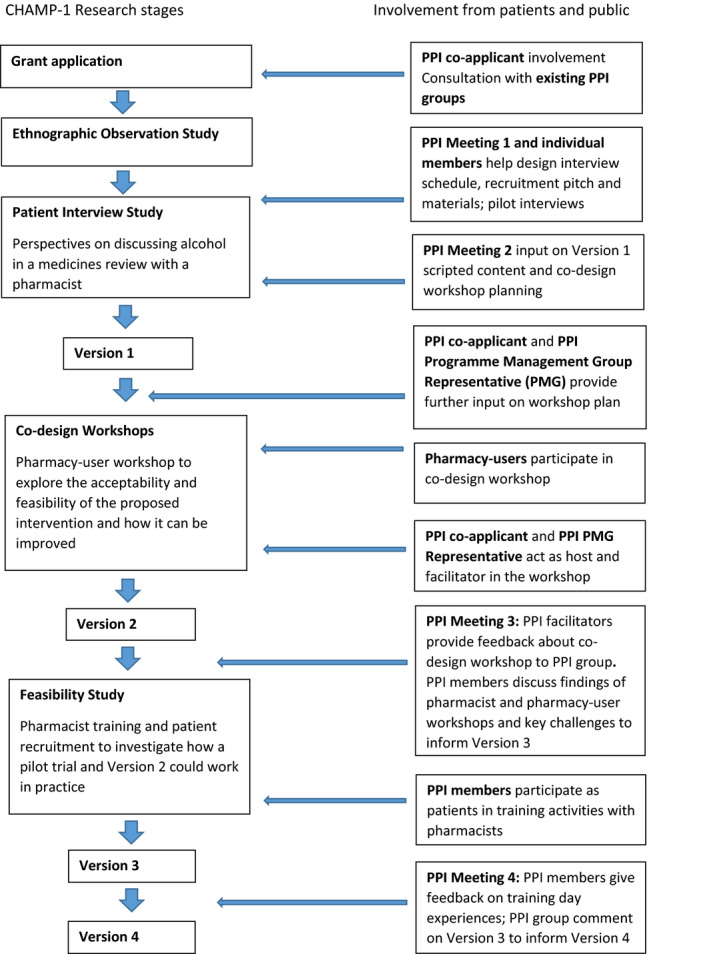
Patient and public involvement input in intervention development phase 1

Our scoping review found that studies of medicine review services focused on the introduction and implementation of the services, with little attention to outcomes for patients.^48^ Consultations were reported to be short and characterized by limited engagement with patients and their health problems. The extent and nature of advice given was rarely examined. In order to learn from both pharmacists and patients about issues and dilemmas that arise in medication (and alcohol) discussions, it was important to get closer to everyday medicines review practice. Exploratory observations found that pharmacists paid little attention to alcohol in the lifestyle section of medicines reviews or elsewhere in practice.^42^ Pharmacists said they found alcohol difficult to raise and to discuss, they felt underprepared and unconfident and that people were reluctant to talk about their drinking. Their main concern was that raising the topic would alienate their customers. These observational findings were discussed with the pharmacy advisory group. This formed the starting point for successive stages of intervention development work. Figure 3 visualizes the PPI involvement in each of the studies and stages of the first‐phase intervention development process, detailed below.

#### 4.3.1 Producing version 1 via qualitative studies

A patient interview study was conducted to better understand the views of people eligible for medicines reviews on the appropriateness of alcohol as a subject for discussion in reviews.^33^ PPI members helped in the design of this study by giving feedback on the interview schedule and by participating in and feeding back on the experience of taking part in pilot interviews. The PPI group also helped to design recruitment materials and a recruitment ‘pitch,’ that is how best to explain the study to potential participants. This input was informed by a PPI group discussion of their own experience of talking about alcohol with health professionals and what made these conversations feel comfortable, useful or the contrary. This included consideration of what it is that influences their own decisions to act on information. The group also discussed the most appropriate terminology to describe people who use the pharmacy, given that community pharmacy is a commercial enterprise which variously uses the terms ‘customer,’ ‘client’ and ‘patient.’ There was no consensus on a standard preferred term because members of the group had issues with all, and with the term ‘pharmacy user.’ The decision was taken to keep the conversation rolling and to tailor the terminology used to the specific context of its use, with a preference for using ‘people’ as far as possible.

The recruitment ‘pitch’ and leaflet produced with the PPI group made the interview study easier to explain concisely to people, and the recruitment target was achieved. This study produced rich data on how people use medicines and alcohol in everyday life, how they use the pharmacy, their experience of medicines reviews and their thoughts on talking about alcohol as part of a medicines review. It found that people were open to the idea of discussing alcohol with community pharmacists in the context of a medicines review if this was sensitively done and was routine, and the relevance was clear to them. Nobody wanted to feel like they were being singled out to talk about their alcohol use.^33^ As in the prior trial that informed the programme bid, many people thought such a discussion was relevant only to those who had a ‘problem’ with alcohol in terms of addiction.^32^, ^33^ They recalled having a pleasant ‘chat’ with a pharmacist but there was poor recall of the content of their medicines reviews and low awareness of their purpose. Version 1 of a new intervention was developed partly based on this work. It was also informed by on‐going analysis of the rationale, context, evidence and methods for intervention development subsequent to the grant application and in discussion with all of the programme advisory groups.

#### 4.3.2 Producing version 2 via a co‐design study

The key components of version 1 of the intervention were discussed with the PPI group along with ways to further develop it in subsequent intervention co‐design workshops which took place with pharmacists and pharmacy users. A key component for discussion was a consultation guide for pharmacists. This included examples of ways to introduce the subject of alcohol comfortably while making its potential relevance clear. Members of the PPI group had a range of responses, especially to the scripted content. Discussion helped to refine this content and emphasized the importance of pharmacy‐user participants at the workshop being able to hear the suggested phrases and consider them as part of an interaction, rather than reducing them to text. How speech was delivered in context would make a big difference to how the intervention was perceived. Perfecting wording without a sense of interaction was inadequate for an intervention grounded in the evidence of ways to support people in exploration of their health, medicines and alcohol use.^49^

A member of the PPI group with design skills suggested the use of a ‘missing piece of the jigsaw puzzle’ symbol to represent alcohol as a missing element in medicines discussions. This was incorporated into the design of the slides used at the pharmacy‐user co‐design workshop. The full details of the design, methods and results of the pharmacy‐user co‐design workshop will be reported separately. The staff team at this workshop included co‐author MO as co‐host and observer, and co‐author DL as a facilitator (having facilitation skills with groups talking about their own alcohol use). DL led one of the subgroups, and research team members led the others. All members of the workshop team commented on drafts of the schedule and facilitators took part in briefing and ‘de‐bugging’ meetings, which further developed working relationships. Recruitment for the pharmacy‐user co‐design workshop was challenging but was helped by building on the success of the PPI group's suggested recruitment recommendations for the patient interview study.

#### 4.3.3 Producing version 3 via a feasibility study

The PPPG and PPI groups met to discuss the findings of pharmacist and pharmacy‐user co‐design workshops to decide whether there was proof of concept, that is, that there was evidence of the acceptability of the intervention, and to make further recommendations for development. A joint meeting was proposed but was not feasible because of on‐going difficulties for pharmacists attending meetings. These were therefore parallel processes where any differences in pharmacist and patient perspectives that arose were discussed by the research team. A key point of difference between pharmacists and people eligible for medicines reviews was that people wanted more information about the service and the opportunity to prepare for it, with an appointment made. However, pharmacists were passionate about the idea of pharmacy as a walk in service, arguing that this was what made pharmacy unique and different from a GP surgery. Arranging appointments was therefore not an option. The PPI group meeting agreed that the co‐design workshops provided proof of concept for the intervention. Ideas from the workshops about follow‐up text messages and supporting leaflets were also discussed. The programme management group made decisions informed by both these processes and other research design and planning considerations.

The next stage was to run a small feasibility study to investigate how trial procedures and version 2 of the intervention could work in practice. Work to date indicated that practice development, including a training component, should play a much larger part of the intervention than had been anticipated (this is separately reported). Two newly recruited PPI members volunteered to be participants in medicines review exercises and gave feedback on this experience during a training workshop for the feasibility study. These members felt pharmacists struggled to raise the subject and to link medicines and alcohol to a person's condition. They reported back to the wider PPI group who agreed that the way the subject of alcohol is brought up is crucial and that this required person‐centred approaches to consultation practice. Most PPI advisors had been interested in self‐empowerment as an important outcome from the outset; others started from the approach that ‘people need telling.’ There is now evidence‐informed agreement in the PPI group that the intervention will need to empower the practitioner in order to empower the person. Comments received on the resulting version 3 of the intervention were used to create version 4, thereby ending the planned intervention development process.

## 5 DISCUSSION

Critics have identified the NIHR approach to PPI and co‐production as highly instrumentalist, positioning these as means to solve a problem and produce a desired outcome.^13^ Complex processes can become abstract ‘things’ to be implemented and managed, decontextualized from the broader social, economic and political contexts in which they operate.^50^ Implementation failure may be attributed to the reluctance of researchers to share power or recognize the value of lay knowledge, experience and skills.^15^ However, the practical challenges involved in co‐producing this programme of research are far more multi‐layered than this, and it is important to acknowledge this if there is to be better understanding of the actual processes by which co‐productive activity occurs.

Funding systems do not allow for meaningful co‐production in advance of research studies beginning, and this places restrictions on how far and in which ways researchers can share power with the groups involved. It remains challenging to fit public involvement activities into the managerial logics and temporal pressures of the health research system and the market‐driven neoliberal university.^51^ There is good reason to question whether the institutional arrangements currently in place can support the ideals of PPI and co‐production as portrayed in the progressive ideas of social movements and to examine which tensions arise and how they play out in practice. The assumptions inherent in the INVOLVE definition may also be worth interrogating further within these contexts. Those championing user‐led research continue to feel marginalized.^52^, ^53^ Space to be flexible and expansive is limited, as are time, outlets and incentives to evaluate and report PPI fully.^4^, ^54^ Being serious about involvement means being serious about evaluation, which requires greater transparency.^55^

CHAMP‐1 is a research programme using a range of research methods and intervention strategies. It seeks to use inputs from co‐production, not for the ‘impression management’^13^ that can result from an imposed funding requirement, but to improve intervention design and further the possibility of achieving person‐centred outcomes, by including the knowledge of those who will deliver and receive the intervention. In our attempt to avoid waste and the ‘dark side’ of co‐production,^2^, ^20^ the strategy has been to operate transparently, opening up a process of continual dialogue while giving critical consideration to the influences that inform the viewpoints and experiences being shared.

## CONFLICT OF INTEREST

The authors have declared that no competing interests exist.

## Data Availability

Data sharing is not applicable to this article as no new data were created or analysed in this study.

## APPENDIX 1

### CHAMP‐1 patient and public involvement meeting impact log

**Table:**

Meeting	Activity description	Impact
Co‐applicant Meeting 29/01/18	PPI co‐applicant helped developed interview guide	Interview guide altered/developed following PPI co‐applicant involvement
PPI Meeting One 22/02/18	Introduction to CHAMP‐1 PPI and Interview Study 2 PPI members participated in pilot interviews and gave interviewers feedback on the interview schedule Helped design flyers to recruit patients to interview study Discussed issues that may affect the consultation with intervention participants Suggested a PPI member role description would be useful	Altered the flyer to contain University of York logo; changed colour scheme and pictures; and altered recruitment question wording Raised initial issues for the research team to consider—for example, feeling judged, ‘told off,’ suggestion of problem drinking PPI member role description made, sent to group and used in recruitment of new members
PPI Meeting Two 23/07/18	Feedback of interview findings; co‐production training; MAC version 1 introduction; and co‐design workshop planning Feedback from researchers: Changes to the flyer and how it was used to recruit people to interviews Interview findings Overview of CHAMP timeline Key take away messages: Alcohol as a drug concept resonated with the group was seen as legitimate for pharmacist and relevant to patients Group suggested pitching alcohol as a drug concept to the workshop participants Members suggested sections of text that did not work well for them One member suggested a ‘Jigsaw’ visual idea for explaining the intervention purpose Discussed options for training—decided training will be incorporated into the meetings and be tailored to the group depending on the timing of the project Signed off on the role description and agreed for all to go through the recruitment process discussion about skills and experience with SM Group said: MAC has potential to build relationships, extending awareness of pharmacist expertise, a health professional ‘on your side’	Gave feedback on written scripted content of MAC guide MAC version 1 no longer included some text, for example ‘in ways that suit them’ Helped researchers work out how to take the MAC guide into the co‐design workshop: The team did not invite comments on written excerpts from the MAC script out of the context. The pharmacist's side of the consultation was read aloud to keep the context clear and to invite discussion of how and when things are said, as well as what is said. The team used ‘alcohol as a drug’ as the overall pitch for the intervention and provided an explanation for it The team used the Jigsaw image in the presentation and in visual aids throughout the workshop

Abbreviations: CHAMP, Community pharmacy: Highlighting Alcohol use in Medication aPpointments; PPI, patient and public involvement.
